# Mind the App: Considerations for the Future of Mobile Health in Canada

**DOI:** 10.2196/15301

**Published:** 2019-11-04

**Authors:** Ma'n H Zawati, Michael Lang

**Affiliations:** 1 Centre of Genomics and Policy McGill University Montreal, QC Canada

**Keywords:** smartphone, mobile phone, regulation, patients, physicians

## Abstract

Over the past decade, smartphone technology has become increasingly sophisticated and ubiquitous. Modern smartphones, now owned by more than three quarters of Canadians and 94% of millennials, perform an array of functions that are potentially useful in the health care context, such as tracking fitness data, enabling health record sharing, and providing user-friendly platforms for disease management. Approximately half of smartphone users have downloaded at least one health app, and clinicians are increasingly using them in their practice. However, despite widespread use, there is little evidence that supports their safety and efficacy. Few apps have been independently evaluated and many lack basic patient protections such as privacy policies. In this context, the demand for the regulation of mobile health apps has increased. Against this backdrop, regulators, including Health Canada, have begun to propose regulating the use of smartphones in health care. In this viewpoint, we respond to Health Canada’s recent proposal to regulate smartphone use in Canada according to a risk-based model. We argue that although Health Canada’s recent proposed approach is promising, it may require complementary regulation and oversight.

## Introduction

As smartphone technology has become increasingly sophisticated and ubiquitous, their use and prominence in health care has grown spectacularly. Modern smartphones, now owned by more than three quarters of Canadians and 94% of millennials [1], perform an array of functions that are potentially useful in the health care context. They are capable of tracking fitness data, enabling health record sharing, and providing user-friendly platforms for disease management. Approximately half of smartphone users have downloaded at least one health app, and clinicians are increasingly using them in their practice [2]. However, despite widespread use, there is an incomplete patchwork of evidence supporting their safety and efficacy [3]. Few apps have been independently evaluated and many lack basic patient protections such as privacy policies [3]. In this context, the demand for the regulation of mobile health apps has increased [4].

## Health Canada’s Draft Guidance

In January 2019, Health Canada issued draft guidance on the regulation of software as a medical device (SaMD). This important development follows early attempts to regulate the proliferation of mobile health apps in the United Kingdom and the United States. The United Kingdom’s National Health Service, for example, has developed an Apps Library, which publishes lists of health apps that have been reviewed on a range of factors, including security and clinical safety [4]. The United States Food and Drug Administration has pursued an approach based on risk assessments, in which apps that function as medical devices and potentially pose risks to patient safety are subject to regulatory oversight [5]. Health Canada’s draft guidance closely follows this risk-based model.

Health Canada’s issuance of the draft guidance on this issue confirms the increasingly prominent role that software plays in health care delivery in Canada and provides clarity on the regulatory status of novel technologies and devices. There are, however, numerous open, persistent questions on the issue of mobile apps that have medical functions. Indeed, for the purposes of Health Canada’s regulatory oversight of mobile health apps under the *Medical Devices Regulations*, the draft guidance specifies that certain mobile apps will be considered SaMD [6]. However, mobile apps meaningfully differ from other kinds of SaMD in at least two respects. First, mobile health apps are incredibly numerous: over 100,000 of them are available on Apple and Android app stores [7]. Second, mobile health apps are ubiquitous: members of the public use them widely, often outside of clinical settings [3]. Each of these factors presents a unique regulatory challenge. Although Health Canada’s recent proposed approach is promising, it will not likely be adequate on its own.

In the draft guidance, software that meets the definition of a medical device according to the *Food and Drugs Act* will be regulated according to a risk-based classification system outlined in the *Medical Devices Regulations*. There are, however, certain kinds of software that Health Canada proposes to exclude from the regulation. Software that matches patient symptoms with treatment guidelines for common illnesses, for example, will not meet the definition of a medical device. Although generally innocuous, such apps may still carry a degree of risk for patients. Consumer apps that draw associations between symptoms and disease might prompt patients to make uninformed health decisions, whereas the collection of personal health information might raise general privacy and data security concerns.

However, potentially deeper challenges exist. Health Canada’s draft guidance proposes to regulate SaMD according to a manufacturer’s *labeled intended use for the product* [6]. In the event of a disagreement on device classification, Health Canada retains final decision-making authority. However, apps will often not have an explicit intended use or may have ancillary functions that approximate a medical purpose even with express indications to the contrary [8]. Furthermore, apps may be made available to the public in the absence of any interaction with Health Canada. The Apple app Store, for example, does not appear to require regulatory clearance for the distribution of medical apps. App Store Review Guidelines specify only that medical apps may be subject to increased scrutiny and that developers should provide documentary evidence of regulatory clearance if such clearance has been obtained [9]. Where an app in public use falls within Health Canada’s regulatory ambit, or where its classification as a medical device is contentious, it is not clear how the agency’s oversight can be managed. Given the number and diversity of available apps, it is almost certain that many of them will fall through the cracks.

Health Canada’s focus on apps as medical devices also potentially obscures that they are increasingly used as tools for conducting health research. Given their unprecedented data collection capacities and ease of distribution, research projects have begun using mobile apps to facilitate investigative work. As they do, the traditionally firm ethical distinction between research and clinical care is becoming significantly more difficult to manage.

## Role of Medical Colleges and Faculties

Taken together, each of these factors underscores the unique complexity of regulating mobile health apps. In fact, mobile health apps may simply be too numerous and too diverse in function to be effectively regulated through 1 government department or agency. Rather, a flexible and multifaceted approach to the regulation of mobile health apps should be pursued. The medical colleges, for one, ought to play an active role in facilitating the use of mobile apps in the clinic. Guidelines for using mobile apps in clinical practice and for recommending them to patients should be developed and periodically revised. Specific limitations on what an app can or cannot do in relation to reserved medical practice should be clarified. Although the Canadian Medical Association has released a guidance document for the prescription of mobile apps by physicians, [10] provincial medical colleges are more directly empowered to regulate the clinical use of health apps among their members, and as mobile health becomes ever more prominent, clinical training that accounts for these trends will become critically important. In parallel, medical education and training programs will play a critical role in preparing physicians to confront the mobile future. Present trends suggest that health apps will be increasingly prevalent features of the Canadian health care system, and physicians must be prepared to understand their powers and limitations. This means that Canadian medical faculties should train future physicians on how mobile apps are likely to affect their practice and their duties to inform, treat, and protect confidentiality and to follow-up. In the future, we may even see the need for a medical computing specialization.

## Conclusions

Whatever approach is ultimately pursued, the medical profession must play an active role in confronting how health apps and other technologies will change the face of health care. This will require a concerted effort by medical colleges to provide both guidance and clinical training to their members. It will also require prospective planning through medical education and, perhaps, specialization. Although an apple a day might keep the doctor away, the same cannot be said, at the moment, of smartphone apps.

## Abbreviations

SaMD: software as a medical device
